# Exploring the Association between Life Perceptions and Emotional Profiles in Taiwan: Empirical Evidence from the National Well-Being Indicators Survey

**DOI:** 10.3390/ijerph17124209

**Published:** 2020-06-12

**Authors:** Mei-Yin Kuan, Jiun-Hao Wang, Yu-Chang Liou, Li-Pei Peng

**Affiliations:** 1 Department of Bio-industry Communication and Development, National Taiwan University, Taipei 10617, Taiwan; mikayla571@gmail.com (M.-Y.K.); wangjh@ntu.edu.tw (J.-H.W.); lipei@ntu.edu.tw (L.-P.P.); 2 Department of Travel Management, JinWen University of Science and Technology, New Taipei 23154, Taiwan

**Keywords:** life perceptions, emotional profiles, subjective well-being, life satisfaction, happiness, worry, depression

## Abstract

Most of the studies on subjective well-being have focused on positive emotions. The adverse effect of negative emotions on mental health has been overlooked. This study investigates the extent to which specific life perceptions are associated with emotional profiles, and explores relevant factors that effectively enhance subjective well-being. The data were drawn from 4656 respondents in the 2015 National Well-being Indicators Survey in Taiwan. T-test, ANOVA, Pearson correlation, and ordinary least squares regression were conducted. The results reveal that perceptions of all life domains are positively associated with life satisfaction and happiness. Depression and worry are negatively associated with most of the life perceptions, except for environmental quality. These results demonstrate that the emotional profile approach sheds light on current literature on subjective well-being, and suggests that strategies to increase well-being should take positive and negative emotion into account simultaneously. The findings contribute by confirming which life domains can produce the best or worst outcomes in emotional regulation and positively influence mental health. Given that personal safety and the future security of external types is the most crucial factor within the emotional profiles, social welfare and protection programs would be an important strategy to increase subjective well-being.

## 1. Introduction

Bhutan’s development experience has recently received increased global attention. Consequently, the United Nations (UN) has included the promotion of subjective well-being (SWB) as one of the key indicators of its Sustainable Development Goals (SDGs) [1,2]. Understanding the causes of SWB has become a major priority for several nations’ development policies [3,4]. At a global level, well-being has become a highly-prized outcome for the purposes of cross-country comparison. For instance, Taiwan was ranked as the happiest country in East Asia and the 26th happiest in the world in the 2018 World Happiness Report [5]. Thus, it is a worthwhile endeavor to investigate the determinants of SWB using nationally representative data from Taiwan, since the results of this investigation can serve as a reference for empirical evidence in many countries.

Following the hedonic approach, the assessment of SWB typically takes into account a series of emotional and cognitive components [6,7]. These two SWB categories are frequently analyzed together holistically. For example, Pavot and Diener [8] have reported that positive emotions, negative emotions, and life satisfaction tend to intercorrelate positively. These factors can also diverge over time and demonstrate different determinants. The distinct nature of positive emotions, negative emotions, and life satisfaction suggests the need to examine these components separately. Understanding variables related to positive emotions, negative emotions, and life satisfaction could potentially result in a more profound understanding of the broader terms of SWB and happiness [9]. However, most prior studies on SWB have focused mainly on discussing single dimensions and the correlation between them [10,11,12,13,14]. Relatively little research has been conducted to assesses positive and negative emotions simultaneously.

The affective components of SWB reflect the balance in a person’s life between pleasant affect and unpleasant affect. Prior studies have found that positive and negative affect correlated differently with several internal and external variables, such as social relationships and life environment [15]. These findings served as the foundation for the distinction between positive affect and negative affect as two separate components of SWB [16]. Subsequently, a number of researchers further demonstrated the distinction between positive affect and negative affect with different correlations. For instance, Steptoe, Deaton, and Stone [17] found that the form and function of positive and negative emotions can be viewed as opposite or complementary, with each tapping into different components of SWB. Other studies have suggested that an adequate assessment of SWB should be composed of at least two inversely associated positive and negative dimensions in the emotional profiles [17,18]. Obviously, individuals’ emotional profiles are related to emotion regulation, SWB, and mental health. Consequently, an emotional profiles approach has the potential to assess people’s well-being more comprehensively.

Another perspective on eudemonic well-being moves away from a focus on happiness towards a focus on human needs. The main idea here is that humans have key psychological needs—i.e., the need to feel a sense of meaning, control, autonomy, and connectedness to others in their lives—and that if these needs are met, the individual can achieve a sense of SWB [4]. The theory of a hierarchy of needs is an example of a theory that conceives of domain satisfactions as needs for SWB [10]. Based on these perspectives, the more needs are satisfied in a given life domain, the greater positive emotion and satisfaction with life individuals will experience as a whole. The eudemonic concept of well-being helps to address the limitations of the hedonic perspective, highlighting that people must satisfy whole life domain needs—and not simply avoid negative emotions and experience happiness—to achieve SWB. Consequently, a number of studies have incrementally suggested the importance of life perceptions in understanding SWB [19,20].

In terms of policymaking, addressing the association between SWB and life perceptions is of particular interest, because the prevalence of mental health problems has risen dramatically in recent years [21,22,23]. Scholars have argued that understanding emotions associated with life perceptions is essential in order to enhance SWB [24,25,26]. In general, life perceptions can be used to ascertain how people assess their lives, leading to an assessment of different life aspects [27]. Previous studies have pointed out that the determinants of SWB and life satisfaction can be identified and evaluated as both internal (personal needs and expectations) and external (specific environmental conditions) types [28,29,30]. Some of these specific domains feature subjective assessments of internal types of determinants—such as personal safety, environmental quality, and future security—while others refer to subjective assessments of external types of determinants—such as health status, personal relationships, and free time allocation.

Prior research has found a correlation between these specific areas and positive and negative emotions and life satisfaction. Regarding internal types, previous studies have implied that health status and personal relationships positively affect SWB [24,31,32,33]. Health status has often been studied in a general sense, and high self-ratings in this category have been associated with high scores on happiness and life satisfaction scales [14]. Additionally, time affluence and allocation are also significantly associated with higher levels of happiness and life satisfaction [31,34,35]. External conditions—i.e., insecurity due to local crime [36] and exposure to airborne pollutants in the environment [34,37]—have been shown to negatively impact the extent to which people in numerous countries appraise the state of their own lives [34]. Howell, Kurai, and Tam [38] indicated that personal safety and future security are prominent factors in determining individuals’ overall well-being [39,40]. In contrast, negative life experiences were found to directly affect mental health and emotions, such as stress, depression, and anxiety [41,42]. In addition, negative emotion is also negatively correlated with life satisfaction [43] and also with general SWB [44]. The relationship between experiencing adverse life events and poor mental condition has also been well-established in the literature [26,45].

These prior studies have emphasized the importance of understanding the roles of socioeconomic characteristics. We expected positive life events, such as marriage, to be related to positive emotions [46]. The attainment of a college education appears to influence how favorably people rate their lives [47]. Prior studies reflect that perceptions in various life domains may lead to positive and negative emotions concurrently. However, when research on SWB focuses solely on the enhancement of positive emotions to improve well-being, the adverse effects of negative emotions on mental health can be overlooked [17,48]. Therefore, a number of studies have suggested that assessing the relationship between multiple life perceptions and emotional profiles might help to confirm the ways in which people increase their SWB [49,50]. In fact, the OECD framework has brought together multiple perspectives on “the most important factor for well-being” [22]. Researchers selected representative indicators—such as health status, social connections, environmental quality, and personal security—on the basis of conceptual soundness and well-established high-quality data. Although this framework has provided helpful guidance, it was designed to be experimental, rather than a rigid set of guidelines [51]. Evidence-based, empirical investigation and verification are required, to validate the importance of indicators in enhancing SWB.

While connections between specific life perceptions and emotions have been investigated, there is still much to learn about the directionality and extent of these relationships. Greater insight into how SWB is supported can potentially reveal how other positive life outcomes may be achieved. Enhancing SWB has been identified as producing higher levels of positive emotion, lower levels of negative emotion, and a high level of general satisfaction with life [7,25]. This highlights the multidimensionality of SWB and reveals certain assumptions about the necessary “conditions” to reach this state (i.e., the enhancement of SWB). For instance, individual factors that can produce higher levels of positive emotions and lower levels of negative emotions simultaneously will be considered important in enhancing SWB. However, few studies have verified these findings. When it comes to policy implications, it is essential to provide information about which aspects of life contribute to individuals’ sense of wellbeing [52]. The present study’s main objective is to investigate the extent to which specific life perceptions are associated with emotional profiles’ measurements of positive and negative emotions. Moreover, this study aims to explore the relevant factors that effectively enhance SWB. Based on these goals, the following hypotheses are proposed: H1a: Internal types of life perceptions are significantly positively correlated with positive emotions; H1b: External types of life perceptions are significantly positively correlated with positive emotions; H2a: Internal types of life perceptions are significantly negatively correlated with negative emotions; H2b: External types of life perceptions are significantly negatively correlated with negative emotions. The results obtained from evaluating these hypotheses are then used to support and verify the determinants of SWB.

The present study is unique in several ways. First, although the importance of SWB has already been recognized, little empirical evidence based on nationwide representative data has been provided thus far. In contrast to earlier studies that relied on the collection of data with limited sample sizes and/or from restricted areas [48,53], this study employed an official dataset from the National Well-Being Indicators Survey. There is a conflict in the existing literature regarding the key factors related to SWB. Due to this ambiguity, studies attempting to examine factors important for SWB rarely investigate the same variables. Most researchers have focused on the study of satisfaction in one individual life domain; for instance, job satisfaction [21]. Given the topic’s multifaceted nature, this study adopted the representative OECD framework to explores the relevance of perceptions within multiple life domains and different aspects of an emotional profile. In addition, prior research has generally been unable to provide clear insights on the determinants of SWB [54]. Therefore, the present study proposes a simple framework employing an emotional profiles approach to measure SWB. The study also provides new insight into the verification of determinants of enhanced SWB.

## 2. Materials and Methods 

### 2.1. Data

The data used in this study were drawn from the 2015 National Well-Being Indicators Survey (NWI), which was conducted by the Directorate-General of Budget, Accounting, and Statistics (DGBAS) of the Executive Yuan (government) in Taiwan [55]. The design for the SWB questionnaire was mainly based on the “Guidelines on Measuring SWB”, published by the Organization for Economic Cooperation and Development (OECD) [22,56]. The Guidelines have been widely used in different cultures/countries, and the Chinese version also has been verified in the research of academia Sinica in Taiwan [55]. The Chinese version questionnaire also adopts a positively-framed question, which is consistent with the English version. The NWI data were collected using a stratified multi-stage survey with Probability Proportionate to Size (PPS) and within-household sampling [55]. After deleting responses with missing values for the key variables, 4656 responses remained to be used in our analysis.

### 2.2. Measurement

#### 2.2.1. Measuring Emotional Profiles

This study focused on the emotional component of SWB. The concept of emotional profiles used in this analysis can be defined as a subjective feeling concerning overall judgments about positive and negative emotions [7]. The study’s questions were derived from the OECD’s Better Life Index, which provides a series of questions to determine respondents’ emotional states—including happiness, worry, and depression—on the previous day [51]. All questions featured an 11-point Likert scale (ranging from 0 = didn’t experience the feeling at all to 10 = experienced the feeling all of the time). Life satisfaction refers to an individual’s overall feelings about life, and it has been identified, in the more recent literature, as one of the ways in which people express positive emotions [57]. In terms of life satisfaction, NWI respondents are asked the following question: “Overall, how satisfied are you with life as a whole these days?” The possible responses are rated on the 11-point scale (ranging from 0 = not at all satisfied to 10 = completely satisfied). According to the previous literature [8,58], we can use life satisfaction and happiness to measure the positive emotional profiles, while worry and depression belong to the negative emotional profiles.

#### 2.2.2. Measuring Life Perceptions

Life perceptions are individuals’ cognitive perceptions about many concrete areas of life, which can be classified into a number of main life domains [8,10]. In other words, life perceptions are evaluations grounded in specific domains, rather than overall satisfaction with one’s entire life [59]. To fulfill this study’s purpose, we selected several life domains that previous research has hypothesized to be associated with positive and negative emotions: internal types (health status, personal relationships, and free time allocation) and external types (environmental quality, personal safety, and future security) [28,60]. The NWI used a set of positively-framed questions asking respondents how they felt about various life domains (for example, the question asks “how satisfied are you with your health status” and “How satisfied are you with your future security”) [51]. All questionnaire questions were designed using an 11-point Likert scale, ranging from 0 = completely disagree to 10 = completely agree.

#### 2.2.3. Socio-Demographic Variables

Previous studies specified several control variables for each respondent including age, educational level, marital status, and gender (=1 if male) [23,58,61]. Detailed definitions and descriptive statistics of all variables are presented in Table 1. Age is further categorized into five groups based on years: 30 and under, 31–40, 41–50, 51–60, and 61 and over. Educational level is categorized into four groups: junior high school or below, senior high school, university or college, and master’s degree or above. In addition, a dummy variable specifies the marital status for respondents who are married (=1 if married).

### 2.3. Statistical Analysis

The empirical analysis was conducted in two steps. First, we explored the relationship between positive and negative emotions and socio-demographic characteristics. We used t-test and ANOVA to calculate differences in various emotions based on socio-demographic characteristics. We also performed a Pearson correlation analysis to calculate the correlations between emotional variables and perceptions of each life domain. Second, an ordinary least squares (OLS) regression method using unstandardized coefficients was employed to examine the relevance of life perceptions in positive and negative emotions. The tolerance and variance inflation factor (VIF) and Durbin-Watson test were calculated to check the multi-collinearity and auto-correlation problem. The estimated emotional profiles equation—including life satisfaction, happiness, worry, and depression—can be specified as:
$Y_{i,l}=\beta_{0}+\beta_{1}D_{i,l}+\beta_{2}X_{i,l}+\varepsilon_{i,l}$(positive emotion, life satisfaction) (1)$Y_{i,h}=\beta_{0}+\beta_{1}D_{i,h}+\beta_{2}X_{i,h}+\varepsilon_{i,h}$(positive emotion, happiness) (2)$Y_{i,w}=\beta_{0}+\beta_{1}D_{i,w}+\beta_{2}X_{i,w}+\varepsilon_{i,w}$(negative emotion, worry) (3)$Y_{i,d}=\beta_{0}+\beta_{1}D_{i,d}+\beta_{2}X_{i,d}+\varepsilon_{i,d}$(negative emotion, depression) (4)
where $Y_{i,l}$ and $Y_{i,h}$ are the levels of positive emotion for life satisfaction and happiness of individual *i*, respectively. $Y_{i,w}$ and $Y_{i,d}$ are the levels of negative emotion for worry and depression of individual *i*, respectively. $D_{i,l},\;D_{i,h},D_{i,w}\;\mathrm{and}\;D_{i,d}$ are the vectors of selected variables for each life domain perception of health status, personal relationships, personal safety, environmental quality, future security, and free time allocation. $X_{i,l},\;X_{i,h},X_{i,w}\;\mathrm{and}\;X_{i,d}$ are exogenous variables for gender, age, educational level, and marital status. The estimated parameters *β* is the vector of parameters to be estimated, and $\varepsilon_{i,l,}\;\varepsilon_{i,h,}\;\varepsilon_{i,w,}\;\mathrm{and}\;\varepsilon_{i,d,}$ represent the random error. All analyses were conducted using the statistical SPSS software (version 22.0, SPSS Inc., Chicago, IL, USA).

## 3. Results and Discussion

### 3.1. Descriptive Statistics of the Sample Characteristics

Table 1 presents the sample statistics of the selected variables. Out of 4656 respondents, 44.6% were male, 44.1% were 31–50 years old, and nearly half graduated from college or university. In addition, 60.9% of respondents were married. In terms of respondents’ perceptions of different life domains, personal safety (7.12 ± 1.952) and personal relationships (6.84 ± 1.928) have the highest scores in this study. In contrast, the life perceptions with the lowest perceived score is future security (6.13 ± 2.277). On average, respondents experienced more positive emotion than negative emotion. For instance, the value for life satisfaction was 6.68 (± 2.069), while the value for depression was 2.31 (±2.600) (see Table 2, final two rows).

### 3.2. Correlations between Emotional Profiles and Life Perceptions

Table 2 presents the results of the correlation matrix among variables. The individual coefficients of emotional profiles are between −0.391 and 0.668. Overall, the two positive and two negative emotions were moderately to largely correlated to each other in meaningful, congruent ways. For example, life satisfaction and happiness were positively correlated with each other, while being negatively related to worry and depression, and vice versa. These results are consistent with previous studies that have demonstrated that the relationship between positive and negative emotion tends to be inversely correlated [6,17]. The present study’s results also indicate that the perceptions of each life domain were significantly inter-correlated with each other. Individual coefficients of life perceptions fall between 0.316 and 0.571.

We continually examined the relationship between life perceptions and emotional profiles and found their individual coefficients to lie between −0.255 and 0.594. A higher score for a life perception was correlated with higher levels of happiness and life satisfaction. In contrast, worry and depression were negatively related to all life perceptions in a significant way. The largest correlation coefficient, with a value of 0.594, was found between future security and life satisfaction. The correlation coefficient between environmental quality and depression represented the lowest score (r = −0.255). These findings are consistent with those of previous studies [32,39], which have suggested that positive emotion is correlated with higher levels of life perceptions, while negative emotion is correlated with lower levels of life perceptions.

### 3.3. Relationship between Emotional Profiles and Life Perceptions Differences in Emotion Based on Socio-Demographic Characteristics

Based on the ANOVA and t-test results (Table 3), most of the socio-demographic characteristics—except for educational level—were significantly different when it came to positive emotion. For instance, female respondents reported higher scores of life satisfaction and happiness than male respondents. In terms of negative emotion, age and marital status were significantly different for worry. The worry scores were higher for respondents in the elderly and unmarried groups. In addition, educational level and marital status were significantly different for depression. Unmarried respondents and those with lower educational levels exhibited higher depression scores than their counterparts. However, a gender difference only appeared with positive emotion, and not negative emotion. The results also reveal that there is a statistically significant age difference in life satisfaction, happiness, and worry, but not depression. This is consistent with Deng, Chang, Yang, Huo, and Zhou [62], who showed that gender differences in emotional experience depend on different types of emotion. We did not uncover any educational difference in either worry or life satisfaction. Finally, a significant marital difference was found for both positive and negative emotions. These socio-demographic differences in various emotions generally replicate prior findings [53,63].

### 3.4. Association between Emotional Profiles and Life Perceptions

Table 4 presents the estimation results of the OLS regression of emotional profiles using unstandardized coefficients. All explanatory variables used in the linear regression models passed the collinearity diagnostics through the variance inflation factor test (all the tolerance values greater than 0.1 and all the values of VIF are less than 10). Then Durbin-Watson test was applied to examine autocorrelation among residues. As a result, all Durbin-Watson value are between 1.5 and 2.5, which meant the presumption of independence of errors is met [64].

We started by examining the association between life perceptions and positive emotion. The results indicate that two types of life perceptions are positively related to respondents’ life satisfaction and happiness levels, indicating that H1a and H1b are supported. The results show that all life perceptions are positively related to respondents’ life satisfaction and happiness levels. These results are consistent with earlier findings in the literature [31,33,65]. Among regression coefficients, future security has the strongest association with life satisfaction (b = 0.251, *p* < 0.001), while personal relationships have the weakest association with life satisfaction (b = 0.066, *p* < 0.001). This finding is in alignment with Abbott et al. [21] and Howell et al. [38], who have argued that reasonable economic, social, and security of external social conditions are highly related factor in terms of life satisfaction. In addition, previous studies have claimed that human relationships another important internal factor in relation to individual happiness [66,67]. Our results confirm that, compared to other perceptions of life domains, personal relationships exhibit the greatest association with happiness (b = 0.150, *p* < 0.001). Environmental quality, for instance, has the weakest association with happiness (b = 0.065, *p* < 0.001).

Overall, these results reveal that different life perceptions have a significant association with positive emotions. The strongest related external factor in terms of life satisfaction was future security (b = 0.251, *p* < 0.001), while personal relationships of internal type have the highest association with happiness (b = 0.150, *p* < 0.001). Ultimately, this study confirms the greatest association with the life perceptions of future security and personal relationships in determining happiness and life satisfaction, which is consistent with previous studies [40,68]. These results also reflect the fact that satisfaction and happiness each capture different dimensions of SWB through the different relevance of internal and external types of life perceptions, respectively [69].

Socio-demographic characteristics are also related to positive emotion. In accordance with the findings of Eren and Aşıcı [70], this study’s results show that being male has a negative association with life satisfaction and happiness, compared with being female. Respondents aged between 31 and 60 years old experienced lower levels of happiness and life satisfaction compared with respondents aged 30 or younger. More specifically, compared to young people, middle-aged people are more likely to have a tendency to suppress positive emotion. As in earlier studies [33,70,71], the present study’s results also suggest a U-shaped relationship between age and happiness. It also appears that individuals who have completed senior high school and above are likely to experience higher life satisfaction and happiness. In addition, consistent with Stutzer and Frey [72], we did find that married individuals have a higher degree of positive life satisfaction than unmarried ones.

We thoroughly examined the association between the life perceptions on negative emotion. On the right side of Table 4, the results show that most of the life perceptions—except for environmental quality—are positively and significantly related to worry and depression. These results are consistent with earlier findings in the literature [41,45]. Our findings support H2a. The H2b hypothesis is that external types of life perceptions are significantly negatively correlated with negative emotions; this hypothesis is partially supported—personal safety and future security are significantly associated with worry and depression, while environmental quality is not.

Among the evaluated domains, personal safety of external types emerged as the strongest related factor in terms of worry (b = −0.239, *p* < 0.001) and depression (b = −0.224, *p* < 0.001). Consistent with the results found by Buonocore, Russo, and Ferrara [73] and Wutich and Ragsdale [42], our findings confirm that individuals with highly insecure perceptions about their external social and environment tend to experience negative feelings. This may also reflect that a lack of freedom from physical and psychological harm might be an underlying trigger for worry and depression.

Some of the socio-demographic characteristics we evaluated also linked to negative emotions. Previous studies uncovered similar findings, indicating that elderly individuals reported relatively lower levels of negative emotion than younger adults [74]. Our results confirm that, compared with the group aged 30 and younger, respondents aged 61+ exhibited negatively associated with worry significantly. Finally, respondents who finished senior high school and university exhibited significantly less depression compared to respondents with lower educational levels. This suggests that higher education might reduce the likelihood of experiencing depression [75,76]. Additionally, our findings are also in alignment with those of Eren and Aşıcı [70], who found no significant gender differences in negative emotion. 

Overall, the life perceptions and socio-demographic characteristics explained 46.1%, 32.7%, 21.0%, and 20.9% of the variations in life satisfaction, happiness, worry, and depression, respectively. As expected, most of the life perceptions that were positively associated with positive emotions and inversely associated with negative emotions, except for the perception of environmental quality. These findings present several noteworthy points. First, our results are in accordance with the well-being literature, suggesting that policymakers must devote greater efforts to enhancing positive emotion and reducing negative emotion [25]. Considering life as a whole, SWB reflects an overall self-assessment of individual experiences across different life domains of perceptions. Therefore, our findings also confirm that perceptions of specific life domains might have different relevance of SWB. Specifically, the internal types (health status and free time allocation) are more related to positive emotions than negative ones. Individuals’ relationships to happiness are mainly determined by life experience in terms of personal relationships. It is worth noting that the external types of future security and personal safety exhibit a high correlation with both life satisfaction and negative emotions (worry and depression). These findings suggest the possibility that external types of life perceptions have a greater impact on well-being than internal types. Such a finding is particularly relevant for public policymakers to enhance subjective well-being through public policies that focus on interventions designed to alter people’s external environments, rather than only focusing on satisfying internal needs.

However, environmental quality was positively associated with positive emotion, but no significant association was found with negative emotion in the regression model. It is evident, though, that environmental pollution negatively related to human health and life satisfaction, as previous studies have shown [34,77]. Our results did not find significant evidence to support the association between perception of environmental quality and the experience of worry and depression. A possible explanation for this might be that the perception of environmental quality has an indirect relation with negative emotion, potentially mediated by perceptions of personal safety and/or future security.

## 4. Conclusions

Although the prior literature has extensively examined the factors associated with SWB and mental health [12,32,41], relatively little information has been uncovered about the association between life perceptions and people’s emotional profiles in terms of SWB. To close this knowledge gap, the present study has investigated the extent to which specific life perceptions are associated with emotional profiles. Using nationwide survey data, the study’s findings identified that different life perceptions are positively associated with life satisfaction and happiness. On the other hand, negative emotions are negatively associated with most of the life perceptions evaluated herein, except for the life domain of environmental quality. As expected, higher levels of most life perceptions were associated with greater personal happiness and life satisfaction, and with less worry and depression. Additionally, in this study, each life perception has a specific relevance regarding the emotional profiles’ distinct components. Specifically, personal relationships of an internal type exhibit the greatest association with happiness, while future security of an external type has the strongest association with life satisfaction. Moreover, personal safety of an external type was strongest associated with worry and depression. These findings provide further evidence that positive and negative emotions might simultaneously contribute to enhancing an individuals’ sense of a harmonious life, without necessarily offsetting each other. This may also reflect people’s capacity to experience mixed emotions.

The study’s findings are particularly relevant for policymaking. Current policy strategies for increasing SWB have focused mainly on positive emotion without paying enough attention to the adverse effects of negative emotion on psychological or mental health. However, separating positive and negative emotion in this way might lead to an imbalance in the development of holistic life well-being. Hence, the emotional profile approach employed in this study sheds light on the current literature on SWB, and suggests that future strategies to increase well-being should take positive and negative emotion into account simultaneously. Policy interventions are likely to be ineffective in enhancing individuals’ SWB, if they do not take into account the effect of both negative and positive emotion.

Overall, the study’s findings revealed that several life perceptions exhibit higher levels of positive emotional outcomes and lower levels of negative emotional outcomes. These results confirm which life domains can produce the best (and/or worst) outcomes in emotional regulation and therefore contribute positively to SWB and mental health [26,48]. Consequently, a number of policy implications can be derived from our results. Given that personal safety and future security of external type is the strongest related factor in the emotional profiles, additional social welfare and protection programs must be established. For instance, implementing programs that address social insurance, healthcare, poverty alleviation, and crime prevention would be a significant strategy to increase individuals’ life satisfaction. Additionally, public policy prescriptions should encourage more leisure activities and resources to facilitate personal interaction and the cultivation of social relationships [34].

As outlined above, this study presents a number of noteworthy findings. However, these findings are based on cross-sectional and retrospective data, which limits inferences about processes and the causality of perceiving emotional profiles. Moreover, this limitation neglects potential bidirectional effects. Longitudinal data that becomes available in the future could be useful for exploring the causality of those relationships. In addition, the use of cross-sectional data does not allow us to identify the effect of individual heterogeneity in preferences and the implications of this for significance testing. We did not correct for multi-testing, since such correction seems less necessary in an exploratory study featuring many variables. It is also possible that the use of multiple testing adjustment limited the study’s discovery of potentially meaningful findings [15]. Second, due to the limitations of using an existing dataset, measurement biases associated with self-reported information could not be overcome in our analysis. Additional objective measures—such as information on monthly income; health status from doctor’s visits; and behavioral measures, in the form of attendance at community work or activities—should be collected to correct this [78]. Further in-depth analyses of domain parallel use of objective and subjective measures would help to better explore associations with emotional profiles, thereby eliminating some of the endogeneity of the self-report questions. Furthermore, the emotional profiles’ measurement criteria were drawn from short emotional scores for measuring large surveys, rather than for constructing a multidimensional scale. Employing an internationally recognized comprehensive scale, such as the “The Scale of Positive and Negative Experience (SPANE)”, will make our study more robust in the future. Finally, although several individual factors were included in the present study, not all possible factors have been assessed. Thus, more research is needed to identify further protective factors (e.g., personality and lifestyle behaviors, geographical conditions, etc.) and to include other factors related to better overall mental health outcomes.

This study suggests several new research directions. Future research can be conducted to explore the trends of the relationship between life perceptions and well-being. For example, additional studies should employ data from the NWI to investigate the impact of life perceptions on emotional profiles over four periods (2013–2016). Studies should also aim to identify specific reasons behind these increasing trends and conduct mediation analyses to elucidate potential pathways. Additionally, future research should use theory and/or natural experiments to uncover latent instrumental variables, and to resolve endogeneity issues. Further studies should also take into account the impact of experiences of neutral or mixed emotions. 

## Figures and Tables

**Table 1:** Socio-demographic characteristics of samples.

Socio-Demographic Characteristics		*n*	%
**Gender**	Male	2078	44.6
	Female	2578	55.4
**Age**	30 years old and below	1293	17.9
	31–40 years old	1084	22.1
	41–50 years old	1012	22.0
	51–60 years old	862	20.6
	61 years old and above	405	17.4
**Educational Level**	Junior high school or below	607	13.1
	Senior high school	1278	27.4
	University or college	2165	46.5
	Master’s degree or above	606	13.0
**Marital Status**	Single or others	1821	39.1
	Married	2835	60.9

**Table 2:** Correlation matrix of the socio-demographic characteristics and emotional profiles.

Variables	1	2	3	4	5	6	7	8	9	10
1. Life satisfaction	−									
2. Happiness	0.548 ***	−								
3. Worry	−0.391 ***	−0.480 ***	−							
4. Depression	−0.399 ***	−0.535 ***	0.668 ***	−						
5. Health status	0.467 ***	0.404 ***	−0.316 ***	−0.317 ***	−					
6. Personal relationships	0.438 ***	0.422 ***	−0.305 ***	−0.320 ***	0.447 ***	−				
7. Free time allocation	0.469 ***	0.400 ***	−0.318 ***	−0.301 ***	0.364 ***	0.375 ***	−			
8. Personal safety	0.529 ***	0.444 ***	−0.385 ***	−0.380 ***	0.487 ***	0.517 ***	0.427 ***	−		
9. Future security	0.594 ***	0.471 ***	−0.385 ***	−0.383 ***	0.507 ***	0.492 ***	0.518 ***	0.571 ***	−	
10. Environmental quality	0.435 ***	0.354 ***	−0.264 ***	−0.255 ***	0.365 ***	0.363 ***	0.429 ***	0.425 ***	0.488 ***	−
*Mean*	6.68	6.93	3.32	2.31	6.69	6.84	6.69	7.12	6.13	6.68
*SD*	2.069	2.096	2.623	2.600	1.934	1.928	2.270	1.952	2.277	2.028

*** *p* < 0.001.

**Table 3:** Relationship between socio-demographic characteristics and emotional profiles.

Demographic Traits		Life Satisfaction			Happiness			Worry			Depression		
		*Mean*	*SD*	*F/t*	*Mean*	*SD*	*F/t*	*Mean*	*SD*	*F/t*	*Mean*	*SD*	*F/t*
**Gender**	Male	6.46	2.15	−6.33 ***	6.74	2.12	−5.40 ***	3.34	2.62	0.45	2.38	2.60	1.67
	Female	6.85	1.99		7.08	2.06		3.31	2.63		2.25	2.60	
**Age**	30 years old and below	6.67	2.01	7.46 ***	6.93	1.91	5.37 ***	3.58	2.60	9.60 ***	2.37	2.57	1.36
	31–40 years old	6.43	1.94		6.77	1.96		3.56	2.60		2.36	2.53	
	41–50 years old	6.66	2.01		6.83	2.17		3.34	2.52		2.39	2.60	
	51–60 years old	6.73	2.14		6.98	2.17		3.15	2.64		2.19	2.59	
	61 years old and above	6.96	2.24		7.19	2.22		2.94	2.73		2.19	2.70	
**Educational Level**	Junior high school or below	6.59	2.54	0.92	6.76	2.52	2.78 *	3.36	2.87	2.08	2.70	2.95	6.97 ***
	Senior high school	6.65	2.09		7.04	2.13		3.21	2.70		2.13	2.64	
	University or college	6.69	1.96		6.91	1.98		3.32	2.55		2.28	2.50	
	Master’s degree or above	6.77	1.85		6.95	1.93		3.52	2.44		2.38	2.31	
**Marital Status**	Single or others	6.43	2.15	−6.49 **	6.74	2.10	−4.99 ***	3.57	2.68	5.10 ***	2.49	2.65	3.91 ***
	Married	6.84	2.00		7.05	2.08		3.16	2.57		2.19	2.56	

* *p* < 0.05; ** *p* < 0.01; *** *p* < 0.001.

**Table 4:** Ordinary least squares (OLS) estimation of regression model (dependent variable: emotional profiles).

Independent Variable	Life Satisfaction	Happiness	Worry	Depression	Collinearity Statistics	
					Tolerance	VIF
Constant	1.045 ***	1.778 ***	8.676 ***	7.626 ***		
**Life perceptions**						
Health status	0.132 ***	0.130 ***	−0.115 ***	−0.112 ***	0.650	1.538
Personal relationships	0.066 ***	0.150 ***	−0.077 ***	−0.106 ***	0.636	1.573
Free time allocation	0.132 ***	0.124 ***	−0.119 ***	−0.100 ***	0.644	1.552
Personal safety	0.187 ***	0.148 ***	−0.239 ***	−0.224 ***	0.553	1.810
Future security	0.251 ***	0.146 ***	−0.167 ***	−0.178 ***	0.486	2.057
Environmental quality	0.096 ***	0.065 ***	−0.027	−0.018	0.681	1.467
**Gender**						
Female (ref.)						
Male	−0.267 ***	−0.224 ***	−0.060	0.020	0.973	1.028
**Age**						
30 years old and below (ref.)						
31–40 years old	−0.177 *	−0.071	−0.224	−0.164	0.479	2.086
41–50 years old	−0.213 **	−0.244 **	−0.180	0.124	0.438	2.283
51–60 years old	−0.327 ***	−0.245 *	−0.208	0.045	0.432	2.316
61 years old and above	−0.173	−0.042	−0.374 **	0.025	0.427	2.340
**Educational level**						
Junior high school or below (ref.)						
Senior high school	0.006	0.246 **	−0.186	−0.497 ***	0.396	2.523
University or college	0.159 *	0.199 *	−0.189	−0.410 ***	0.339	2.954
Master’s degree or above	0.281 **	0.269 *	0.026	−0.284	0.482	2.074
**Marital status**						
Single or others (ref.)						
Married	0.156 **	0.113	0.012	−0.034	0.677	1.477
*R*	0.679	0.572	0.459	0.457		
*R^2^*	0.461	0.327	0.210	0.209		
*F values*	264.884	150.479	82.391	81.733		
Durbin-Watson	1.978	1.981	1.994	2.032		

* *p* < 0.05; ** *p* < 0.01; *** *p* < 0.001.
